# The effect of a hydro-alcoholic extract of olive fruit on reproductive argons in male sprague-dawley rat

**Published:** 2013-04

**Authors:** Parvaneh Najafizadeh, Farzaneh Dehghani, Mohammadreza Panjeh Shahin, Sommaye Hamzei Taj

**Affiliations:** 1 *Department of Pharmacology, Medical School, Shiraz University of Medical Sciences, Shiraz, Iran.*; 2 *Histomorphometry and Stereology Research Center, Department of Anatomy, Medical School, Shiraz University of Medical Sciences, Shiraz, Iran.*; 3 *Medicinal and Natural Products Chemistry Research Center, Medical School, Shiraz University of Medical Sciences, Shiraz, Iran.*; 4 *Department of Physiology, Medical School, Shiraz University of Medical Sciences, Shiraz, Iran.*

**Keywords:** *Spermatogenesis*, *Infertility*, *Phytoestrogens*, *Olea Europaea*

## Abstract

**Background: **Olive (*Olea europea*), from the Oleaceae family, is known as a phytoestrogen plant compound, containing Lignans and phenoliccompounds. Some studies have shown phytoestrogens to have spermatogenesis-decreasing effects.

**Objective:** The present study investigated the effects of a hydro-alcoholic extract of olive fruit on reproductive argons in male rats.

**Materials and Methods: **The hydro-alcoholic olive (*Olea europaea*) extract was given orally to three experimental groups of rats in 50, 150, and 450 mg/kg in 48 days. The vehicle group was fed with normal saline and nothing was given to the control group (each group with 8 rats). After 49 days reproductive indicators i.e., sperm count, sperm motility, the weight of prostate, testis, epididymis, and seminal vesicle were measured.

**Results:** The results showed a significant decrease in the weights of the left testicle, seminal vesicle, testosterone hormone, sperm count and sperm motility but there was no significant difference with regard to the weights of prostate and epididymis, and estradiol hormone**.**

**Conclusion:** This study suggests that olive extract may have deleterious effects on fertility factors; therefore, after further studies, it may be used as a contraceptive in males.

## Introduction

Phytoestrogens plant compounds with biologic-estrogenic activity, structurally similar to 17β-estradiol, are first converted to heterocyclic compounds similar to estrogens in structure and then conjugated in the liver (1-3). Phytoestrogens are categorized into three major classes: Isoflavones, Lignans, and Coumestans (4). These plants are vastly available in food sources like soybean, flax seed, fennel and Actinidia chinensis  (5). Epidemiological studies show that food sources containing phytoestrogens cause lower risk of cardiovascular diseases and also prostate and breast cancers (6).

Australian pastures developed a widespread infertility in the 1940s. A particular type of clover (*Trifolium species*) , rich in formononetin, is included in the sheep grazing which in the rumen during the process of fermentation will be changed to daidzein (7). Other studies claim that the phytoestrogens present in a type of summer grass reduced the reproduction rate of sparrows and deer in California; these studies also report that young mice fed by their mothers suffered from infertility problems because they were exposed to high amounts of phytoestrogens (8-10). It was also observed that soy bean caused infertility in Cincinnati’s panthers, a problem solved by eliminating soy bean from the food supply (11).

Olive (*olea europea*), from the oleaseae family, is known as a phytoestrogen plant compound since it contains Lignans and phenolic compounds (12-14). Olive contains stilbenoids, phenolic acidand flavonoids, and, because of the presence of oleuropein, has antioxidant, anti hyperlipidemic and anti-ischemic effects (15). It is also useful in curing gastrointestinal problems since it has laxative effects (16). What’s more, olive is employed in treating dermatological diseases like psoriasis and atopic dermatitis (17). 

Additionally, the plant has antimicrobial, antivirus and anti-fungus attributes (18-19). It should be mentioned that olive reduces osteoporosis in Menopausal women (20). Therefore, with regard to the phytoestrogenic effects of this plant, the present study investigated the effects of olive extract on the fertility reduction of male rats.

## Materials and methods

**Plant collection and preparation of extract**

Olive fruit was collected in summer from Kesht-o-Sanate Bayza Co., (Shiraz, Iran) and the class was specified by an expert to be Olea Europaea L (Voucher Number: 037422) (21). Then the fruit supply was dried in exposure to air and away from sun beam, and after being crushed, was taken to the percolator where it was percolated by means of ethanol 70% (4 times per day, 20cc solvent each time, for 25 days). The resultant ethanol extract was preserved in closed and dark containers in refrigerator until the time of experiment.

**Animals treatment**

In this experimental study, 40 Sprague-Dowley male rats with the average weight of 200-250 grams and age of 8-10 weeks, divided into 5 groups (Table I). They were kept at the Animal Center of the Shiraz University of Medical Sciences at a temperature of 26±2^o^C, a cycle of 12h/12h light/dark. They had access to food and water ad libitum for 49 days. The study adheres to the principles of laboratory care established by Ethics Committee of Shiraz University of Medical Sciences.

Before the administration of the first gavage and 24 hours after that of the last one (i.e., in the 49^th^ day), all the rats were weighed, and blood samples were taken from their tail vein. The blood samples were then centrifuged (1500 rpm, 20 minutes),the serum was separated, and stored at -80^o^C for the measurement of estradiol and testosterone, using immunoassay technique. Spectra Testosterone, and estradiol kits were used according to their manufacturer’s instruction (Orion Diagnostica; Finland and DRG Instruments GmbH; Germany). 

In the 49^th^ day and under anesthesia by ether, the rats were dissected and the reproductive organs including the left testicle, epididymis, seminal vesicle and the left prostate were removed and cleaned by physiological serum. After removing lipid remnants, the organs were weighed by a digital scale, and the exact measures were recorded for the following analyses.

**Sperm motility**

Animals were sacrificed and their reproductive organs were dissected; a length of 1cm of the left end of vas deferens duct was horizontally cut. The location was chosen because of the presence of more mature sperm cells in comparison to the beginning area of the duct (22). The sperm cells were then placed in 5ml of Hanks Balance Salt Solution (HSBB) on the incubator set at 37^o^C so that they were evenly distributed. 

Then, 250µl of the liquid was taken by a sampler and the motility was measured under a microscope with a magnifying power of 40X as follows: ten spots were randomly chosen; in each, the sperm motility was monitored and measured as one of Grade a (these are the strongest of sperm cells and swim fast in a straight line; sometimes it is also denoted motility 1); Grade b (these also move forward but tend to travel in a curved or crooked motion; sometimes also denoted motility 2); Grade c (they do not move forward despite the fact that they move their tails; sometimes also denoted motility 3); or Grade d (these are static and fail to move at all; sometimes also denoted motility 4) (23).

**Sperm count**

The sperm samples present in the Hanks medium were loaded on the neubauer hemocytometer for counting the sperm numbers. Then, the sperm count of 1 mm^3^ of diluted semen was computed by Equation 1:

$\left(1\right)A=\mathrm{B.C.D}$

where A and B stand for the total sperm count taken from 1cm of vas deferens, the sperm count of 0.1mm3 of the liquid, respectively, and C and D equal 10 and 5000 mm as the depth and concentration factors, respectively (24).

**Statistical analysis**

Quantitative data are presented as Mean±SD. Sperm count and motility, of control and experimental groups are compared using one-way analysis of variance (ANOVA), and Tukey test is use to find the statistical differences among their means. P<0.05 is considered to be statistically significant.

## Results

**Figure 1:**
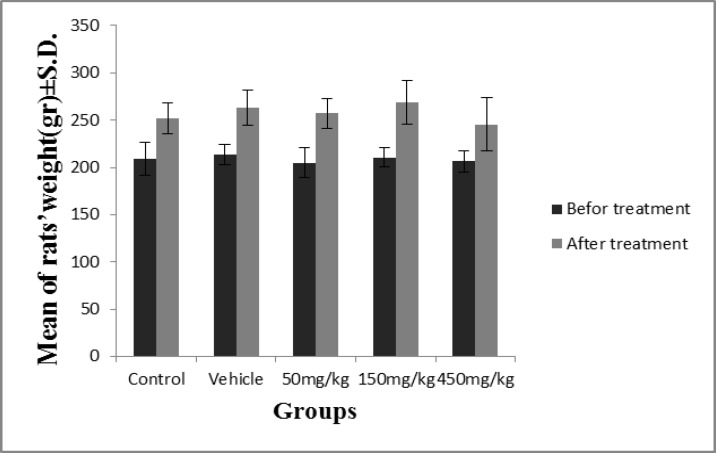
The effect of different dosages of olive extract on rats’ weights. There was no significant difference in the rats’ weights among the control, the vehicle, or the experimental groups

Oral administration of various concentrations of olive extract resulted in no significant difference in the rats’ weights among the control group, the vehicle, and the experimental groups (Figure 1). The weights of the left testicle in the groups administered dosages of 50, 150, and 450 mg/kg and seminal vesicle in the groups administered a dosage of 150 mg/kg showed a significant decrease (p=0.03). 

**Figure 2:**
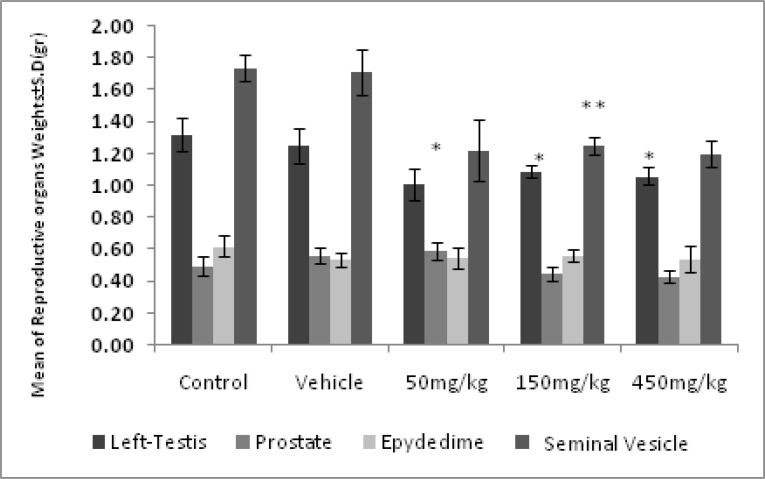
The effect of the olive extract on the weights of prostate, seminal vesicle, left testicle and epididymis. * There was a significant decrease in left testis’ weight in the experimental groups compared to the control and the vehicle groups. ** There was a significant decrease in Seminal Vesicle weight in the experimental groups compared to the control and the vehicle groups

**Figure 3:**
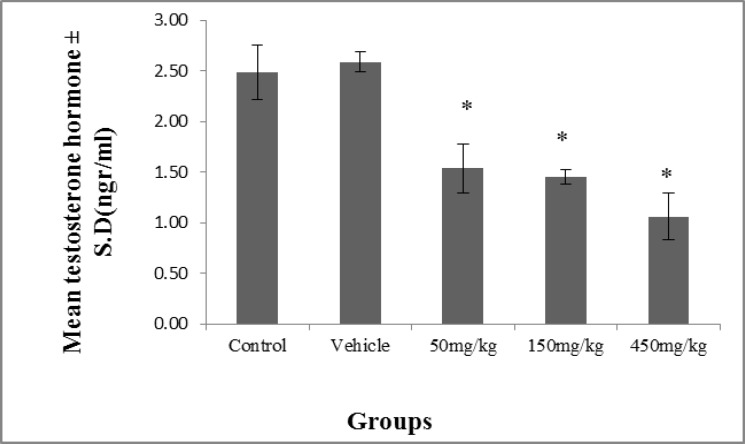
The effect of olive extract on testosterone levels (ngr/ml). * There was a significant decrease in testosterone levels in the experimental groups compared to the control and the vehicle groups

However, there was no noticeable difference with regard to the weights of prostate (p=0.07) and epididymis (p=0.10) (Figure 2).The results of the measuring of the testosterone demonstrate a significant decrease (p≤0.04) in testosterone in the experimental groups in comparison with the control group. The highest decrease was observed in the group administered the 450 mg/kg dosage (Figure 3). 

**Figure 4:**
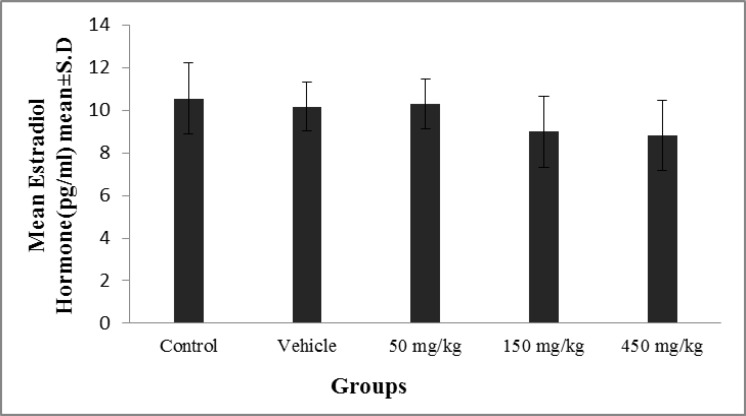
The effect of olive extract on estradiol levels (pgr/ml).There was no significant difference in the rats’ weights among the control, the vehicle, or the experimental groups

The results of the measuring of the estradiol, reveal no significant difference among the control, vehicle and/or other experimental groups (p≤0.07) (Figure 4).

**Figure 5:**
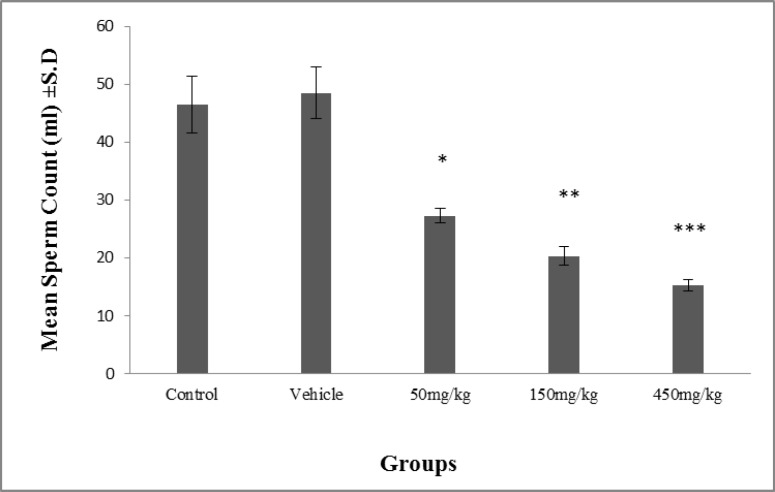
The effect of the olive extract on sperm count. There were significant dosage dependent decreases in the experimental groups compared to the control and the vehicle groups. The decrease enhanced as the dosage level increased. * There was a significant decrease in sperm count of the experimental group treated with a dosage of 50 mg/kg compared to the control and the vehicle groups. ** There was a significant decrease in sperm count in the experimental group treated with a dosage of 150 mg/kg compared to the experimental group treated with a dosage of 50 mg/kg, the control and the vehicle groups. *** There was a significant decrease in sperm count in the experimental group treated with a dosage of 450 mg/kg compared to the experimental groups treated with dosages of 50 and 150 mg/kg, the control and the vehicle groups

There was a significant decrease (p≤0.001) in the sperm count of the groups administered dosages of 50, 150 and 450 mg/kg/day in comparison with the control and vehicle groups; the most effective dose was 450 mg/kg/day (Figure 5). 

**Figure 6:**
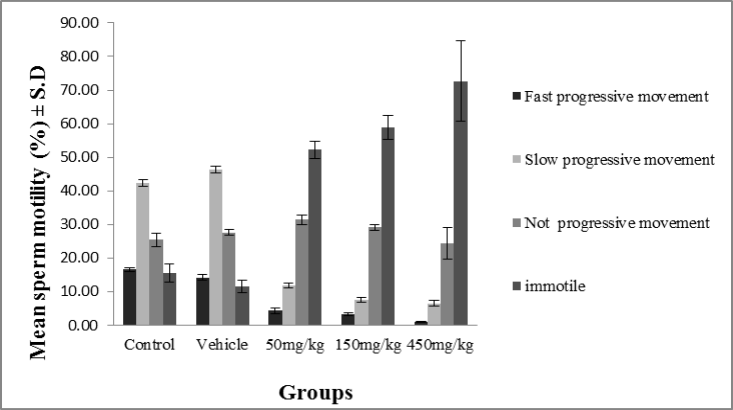
The effect of olive extract on sperm motility in different groups

The results of the study of sperm motility show a significant decrease (p≤0.04) in the sperm motility of the groups administered dosages of 50, 150 and 450 mg/kg/day in comparison with the control and vehicle groups (Figure 6).

**Table I:** Summary of experimental groups and the diet/drug treatment protocols^a^

**Groups**		**Diet therapy**
Control		No
Vehicle		normal saline (1cc)
Experimental groups		
	Experimental group (1)	50 mg/kg/day of the olive extract (1cc)
	Experimental group (2)	150 mg/kg/day of the olive extract (1cc)
	Experimental group (3)	450 mg/kg/day of the olive extract (1cc)

^a^) Each groups consisted of eight rats. ^b^) Medication was through oral administration and through gavage which lasted for 48 days

## Discussion

Phytoestrogens are plant compounds with structures and functions similar to those of 17-β estradiol, which produce effects like those by estrogen (3). The olive, as it contains phenol compounds, is one of the natural plants rich in phytoestrogens, and belongs among the Lignans (13-14). The plant can highly decrease menopausal syndrome in women (25). It also decreases the occurrence of colorectal, prostate and breast cancers (6). The findings of the present study show that olive decreases the levels of reproductive indicators such as sperm count and motility, testosterone, the weights of testicle and seminal vesicle in male rats. The results of the study showed no change in the rats’ weights; therefore, it can be concluded that the extract produces no effect on metabolism. 

The results also show a significant decrease in testosterone level among the five groups, which is dependent on the concentration of the extract; the decrease in testosterone is positively correlated to the concentration of the extract. Studies by Webber *et al* and Roberts *et al* on the effects of phytoestrogens on testosterone support these results. McGravy *et al* found that the LH level in rats decreases as a result of exposure to Genistein. According to their study, it is possible that Phytoestrogen has an inhibitory effect on the enzyme 17 β-hydroxy steroid hydrogenase human type 5; therefore, the synthesis of testosterone in adrenal cortex reduced (27-29)

The results of the study show no significant differences of estradiol levels among the groups. Although, studies by Webber *etal *and Glazier and Boman have also shown that phytoestrogens produce no significant decrease in estradiol levels, and a study in 2005 about the effect of Actinidia Chinensis on male rats’ spermatogenesis showed an increase in estradiol (27, 30, 31). It should also be noted that Actinidia Chinensis belongs to Genisteins while olive is from Lignan group, which can justify the discrepancy of the results of different experiments as a result of the different types of phytoestrogen under study and the differences in the concentrations employed.

The administration of the olive extract in all the three concentrations resulted in a significant decrease in both sperm count and sperm motility. Roberts *et al* also reported similar results (29). One study reported that Genestein phytoestrogen inhibit tyrosine kinase enzymes, which accordingly results in the decrease of sperm count and sperm motility (32). Another study on fennel and Actinidia Chinensis showed similar results (31). On the other hand, there are some studies which have claimed that a phytoestrogen-based regiment has no effect on the quality of mature sperm cells (33, 34). All in all, it can be said that the way a phytoestrogen affects sperm quality depends on its type.

Our findings show significant decreases of the weights of the left testicle and seminal vesicle in three of the administered dosages, but no significant difference in the weights of the left epididymis and prostate. A study by Sprando* et al *showed a decrease in the weights of testicle and seminal vesicle. It should be noted that flax seed is from the Genisteins family (35). Different studies; however, have reported the effects of phytoestrogens on the weights of reproductive organs differently (36). 

With regard to the explained results, there is this possibility that the different effects of phytoestrogens on the male productive system is due to estrogenic and anti-estrogenic effects, as phytoestrogens function through estrogen receptors which have both agonistic and antagonistic properties. Depending on the type of phytoestrogen and the location, the effects can differ. For example, Isoflavones are very weak agonists which bind to estrogen receptors less than estradiol does (37). 

When estradiol levels are low in the body and binding is therefore less competitive, Isoflavones show stronger agonistic effects. On the other hand, the anti-estrogenic effects of Isoflavones are co-dependent on relative concentrations of endogenous phytoestrogens and estrogens, and it is quite possible that when estrogen is high, phytoestrogens make estradiol receptors unavailable to estradiol. Genisteins can also have both estrogen-like and anti-estrogen-like properties (because of the competitiveness in binding to proteins) (37). 

Phytoestrogens produce various physiological effects in both the human body and animal models. Their effects on the male reproductive system depend on the type of the phytoestrogen, concentration and the model under study (17).

## Conclusion

In conclusion, olive fruit extract significantly decreased fertility parameters in the male adult rat. However, it is needed more study about the mechanism by which olive fruit extract creates its anti-fertility effects on human being which are still unknown. Nevertheless, considering our findings in this animal model, it is recommended that the olive fruit extract maybe used in the future as a contraceptive in males.
